# Scaling Concepts in Serpin Polymer Physics

**DOI:** 10.3390/ma14102577

**Published:** 2021-05-15

**Authors:** Samuele Raccosta, Fabio Librizzi, Alistair M. Jagger, Rosina Noto, Vincenzo Martorana, David A. Lomas, James A. Irving, Mauro Manno

**Affiliations:** 1 Institute of Biophysics, National Research Council of Italy, via Ugo La Malfa 153, 90146 Palermo, Italy; samuele.raccosta@ibf.cnr.it (S.R.); fabio.librizzi@cnr.it (F.L.); rosina.noto@cnr.it (R.N.); vincenzo.martorana@cnr.it (V.M.); 2 UCL Respiratory, University College London, 5 University Street, London WC1E 6JF, UK; alistair.jagger.14@alumni.ucl.ac.uk (A.M.J.); d.lomas@ucl.ac.uk (D.A.L.); j.irving@ucl.ac.uk (J.A.I.); 3 Institute of Structural and Molecular Biology, University College London, Gower Street, London WC1E 6BN, UK

**Keywords:** serpins, serpin polymers, atomic force microscopy, dynamic light scattering, conformational disease, polymer theory

## Abstract

$\alpha_{1}$-Antitrypsin is a protease inhibitor belonging to the serpin family. Serpin polymerisation is at the core of a class of genetic conformational diseases called serpinopathies. These polymers are known to be unbranched, flexible, and heterogeneous in size with a beads-on-a-string appearance viewed by negative stain electron microscopy. Here, we use atomic force microscopy and time-lapse dynamic light scattering to measure polymer size and shape for wild-type (M) and Glu342→Lys (Z) $\alpha_{1}$-antitrypsin, the most common variant that leads to severe pathological deficiency. Our data for small polymers deposited onto mica and in solution reveal a power law relation between the polymer size, namely the end-to-end distance or the hydrodynamic radius, and the polymer mass, proportional to the contour length. We use the scaling concepts of polymer physics to assess that $\alpha_{1}$-antitrypsin polymers are random linear chains with a low persistence length.

## 1. Introduction

The serpins (SERine Protease INhibitors) comprise a superfamily of proteins that are primarily inhibitors of serine or cysteine proteases [1]. Their inhibitory mechanism uses an exposed “reactive center loop” (RCL) as a bait for the target protease. When cleaved, the RCL inserts into the main protein β-sheet as an additional strand, translocating the protease [2]. In the resulting complex, the protease is held essentially irreversibly in a partially distorted conformation. The conformational change central to this mechanism is rapid and efficient and utilises the metastability of the serpin native state with respect to the ground-state cleaved conformation [3]. The stability-function trade-off inherent in the serpin fold means it is particularly susceptible to point mutations, and many dysfunctional variants are known. This is the origin of a class of genetic conformational diseases, called serpinopathies [4]. These can exhibit a loss-of-function phenotype due to a deficiency in the levels of active serpin, such as found in hereditary angioedema caused by deficiency of the C1 inhibitor [5], and/or a toxic gain-of-function phenotype due to the deposition of serpin long-chain oligomers (polymers) in tissue, as in the case of familial encephalopathy with neuroserpin inclusion bodies (FENIB; caused by the polymerisation of neuroserpin) [6,7].

An archetypal case is that of $\alpha_{1}$ antitrypsin ($\alpha_{1}$AT), a highly expressed plasma protein for which the primary role is to protect the lungs from proteolysis by neutrophil elastase during an inflammatory response [8]. In $\alpha_{1}$AT deficiency, pathological mutants induce the formation of polymers that accumulate at the site of production in the liver [9], with a concomitant depletion of circulating monomeric $\alpha_{1}$AT levels in the plasma [10]. This accumulation predisposes individuals to liver disease and the deficiency to emphysema.

While many studies have investigated the polymerisation pathway both for $\alpha_{1}$AT [11,12,13,14,15,16,17,18], and for other serpins [19,20,21,22], many details of the formation and the structure of serpin polymers more generally remain lacking or contentious. In particular, there is growing consensus about the existence of different structural polymer morphologies that can be accessed by different pathways [13,20,21,23].

Of the known pathological variants of $\alpha_{1}$AT, the Z mutation (Glu342→Lys) is the most common. Z $\alpha_{1}$AT polymers form and accumulate in the liver [9] and are detectable in the circulation of both homozygous and heterozygous individuals [24]. Wild-type antitrypsin (M $\alpha_{1}$AT) is not polymerisation-prone but can be induced to form polymers by heating [11] or incubating in the presence of a denaturant [9]. Only heat induces a polymer form that shares an epitope also found in Z pathological polymers [13,23,25].

In the present work, we perform Atomic Force Microscopy (AFM) imaging on both Z and M $\alpha_{1}$AT polymers, purified from patients and formed by heating, respectively. As a nano-scale technique, AFM has a sufficient resolving power to achieve a statistically relevant measure of the dimensions and length distribution of monomers and small polymers and to provide structural information of polymer aggregates. The application of classical concepts from polymer physics [26] shows both M and Z $\alpha_{1}$AT polymers to be highly flexible linear chains analogous to random polymers in a good solvent. The self-similarity of polymers of different sizes is revealed by AFM imaging as well as by polymerisation kinetics monitored by static and dynamic light scattering (LS).

## 2. Materials and Methods

### 2.1. Proteins and Reagents

Wild-type (M) monomers and pathological (Z) polymeric $\alpha_{1}$AT were purified from human plasma using Alpha Select resin followed by Q Sepharose chromatography (GE Healthcare, Little Chalfont, UK) as described elsewhere [16] and buffer-exchanged into 10 mM Na_2_HPO_4_ and 100 mM NaCl, pH 7.4, before storage at −80 °C. Protein concentration was determined using an extinction coefficient at 280 nm of 27,000 cm${}^{-1}$ M${}^{-1}$ and a molecular weight of 52 kDa. All chemicals were analytical grade.

### 2.2. Polyacrylamide Gel Electrophoresis (PAGE)

Monomer and polymer $\alpha_{1}$AT (loaded at a final protein amount of approximately 3 μg) were resolved by 7.5% *w*/*v* non-denaturing polyacrylamide gel electrophoresis (PAGE) and stained with PageBlue protein staining solution (Thermo Scientific, Waltham MA, USA) [27].

### 2.3. Atomic Force Microscopy (AFM)

Z-$\alpha_{1}$AT polymer samples at 5.7 mg/mL (purified from plasma) and M-$\alpha_{1}$AT polymer samples (formed by heating a 2.0 mg/mL solution at 70 °C for several hours) were diluted 1:1000 in 10 mM phosphate buffer, pH 3.5. A 40 μL drop was deposited on a freshly cleaved mica substrate, incubated at room temperature for 2 h, and then gently washed with buffer. Images of size 1 μm × 1 μm were recorded using a JPK Nanowizard 3 (Bruker Nano GmbH, Berlin, Germany) with scan rate 2 Hz (1024 × 1024 resolution) and scan rate 1 Hz (512 × 512 resolution) for Z-$\alpha_{1}$AT and M-$\alpha_{1}$AT, respectively. The instrument was operated in liquid tapping mode using AC40 cantilevers (resonance frequency 33 kHz in buffer), equipped with silicon tips with a nominal radius of curvature below 10 nm (the effective radius of curvature was 7 nm and 10 nm for Z-$\alpha_{1}$AT and M-$\alpha_{1}$AT, respectively).

### 2.4. Static and Dynamic Light Scattering (LS)

Time-resolved light scattering experiments were performed at different concentrations of M $\alpha_{1}$AT. Monomeric solutions were filtered (0.2 μm cutoff) directly into a quartz cuvette and incubated at 55 °C or 70 °C in a thermostated cell compartment of a BI200-SM goniometer (Brookhaven Instruments) equipped with a 532 nm solid-state laser. Scattered light intensity and its time autocorrelation function $g_{2}\left(t\right)$ were measured simultaneously by using a BI-9000 correlator (Brookhaven Instruments, Holtsville, NY, USA). Absolute values for scattered intensity (excess Rayleigh ratio $R_{ex}$) were obtained by normalisation with respect to toluene: $R_{ex}=\left(I_{S}-I_{B}\right)/I_{T}*{\left(n_{S}/n_{T}\right)}^{2}*R_{T}$, where $I_{S}$, $I_{B}$, and $I_{T}$ are the scattered intensity of the sample, the buffer, and the toluene, respectively, $n_{S}$ and $n_{T}$ are the refractive indices of the sample and toluene at 532 nm ($n_{S}=1.3367$ and $n_{T}=1.4996$), and $R_{T}$ is the toluene Rayleigh ratio ($R_{T}=28\times 10^{6}\;{\mathrm{cm}}^{-1}$) [22]. The excess Rayleigh ratio is normalised by the mass concentration *c* and the monomer mass $M_{0}$: $R_{ex}/\left(KcM_{0}\right)$, where $K={\left[4\pi n_{S}\left(dn_{S}/dc\right)\lambda^{-2}\right]}^{2}N_{A}^{-1}$. The factor K depends upon Avogadro’s number $N_{A}$, the laser wavelength (λ=532 nm), the refractive index of the medium ($n_{S}=1.3367$), and the refractive index increment $dn_{S}/dc=0.18\;{\mathrm{cm}}^{3}\;g^{-1}$. The normalised Rayleigh ratio is proportional to the weight average mass of polymeric species $M_{w}$ times the z-averaged form factor P(q): $R_{ex}/\left(KcM_{0}\right)=M_{w}/M_{0}\cdot P\left(q\right)/P_{0}\left(q\right)$, where $P_{0}\left(q\right)$ is the form factor of a monomeric protein, that is $P_{0}\left(q\right)=1$ along the experimental scattering vectors [28]. The correlation functions $g_{2}\left(t\right)$ were analysed by using the cumulant method [29]: In$\left[g_{2}\left(t\right)-A\right]/2=\sum_{n}{\left(-1\right)}^{n}{\left(n!\right)}^{-1}k_{n}t^{n}$, where $k_{n}$ is the *n*th cumulant of the relaxation time distribution. The first cumulant $k_{1}=Dq^{2}$ is proportional to the average diffusion coefficient *D* [28]. The average hydrodynamic radius $R_{h}$ is calculated by using the Stokes–Einstein relation: $D=k_{B}T/\left(6\pi \eta R_{h}\right)$, where $k_{B}$ is Boltzmann constant and η is the medium viscosity. The second cumulant $k_{2}$ is the variance in the distribution, and the polydispersity index (PDI) is estimated by the normalised variance $\sigma^{2}=k_{2}/k_{1}^{2}$. The cumulant analysis was performed up to the fourth cumulant, hence limiting data truncation to guarantee a robust estimation of $k_{1}$ and $k_{2}$ [30]. As an alternative approach, intensity autocorrelation functions were fitted by the central moment analysis (up to n=2 or n=3) [31]: $g_{2}\left(t\right)-A=e^{-2\mu_{1}t}{\left[\sum_{n}{\left(-1\right)}^{n}{\left(n!\right)}^{-1}\mu_{n}t^{n}\right]}^{2}$, where $\mu_{n}$ is the *n*th central moment of the relaxation time distribution (for *n* = 1,2,3 $\mu_{n}=k_{n}$). The latter method allows us to obtain reliable results even for high PDI values [30,31].

## 3. Results and Discussion

Z $\alpha_{1}$AT isolated from patient plasma and M $\alpha_{1}$AT polymers induced by moderate thermal stress were visualised by non-denaturing polyacrylamide gel electrophoresis (PAGE). For both samples, a characteristic [27] ladder-like pattern was evident, consistent with a heterogeneous mixture of monomer and polymer chains of variable length (see the Supplementary Materials Figure S1).

In order to image the monomers and polymers, the samples were deposited on mica for AFM imaging. A considerable preliminary effort was used to identify a suitable condition that would promote polymer adhesion to the substrate. Different experimental parameters and procedures were tested including variation in the incubation time on substrate, the extent of sample dilution, different salt concentrations achieved by buffer exchange/dilution, washing or not washing after deposition, and drying. The outcomes were not satisfactory in terms of deposition efficiency and sample adherence. Functionalisation of the substrate by (3-Aminopropyl)triethoxysilane (APTES), gluteraldehyde or magnesium salts was effective at adherence but did not give a completely flat surface free from possible artefacts considering the small size of polymer aggregates. The pI of $\alpha_{1}$AT is 5.8 and the Gordian knot was solved by diluting polymers in an acid buffer (pH 3.5) in order to confer them with a net positive charge for adherence on negatively charged mica. As polymers are already mature and morphologically stable to chemical denaturation [32], it is reasonable to assume that the buffer exchange does not modify their shape and their bulk structural properties.

**Figure 1:**
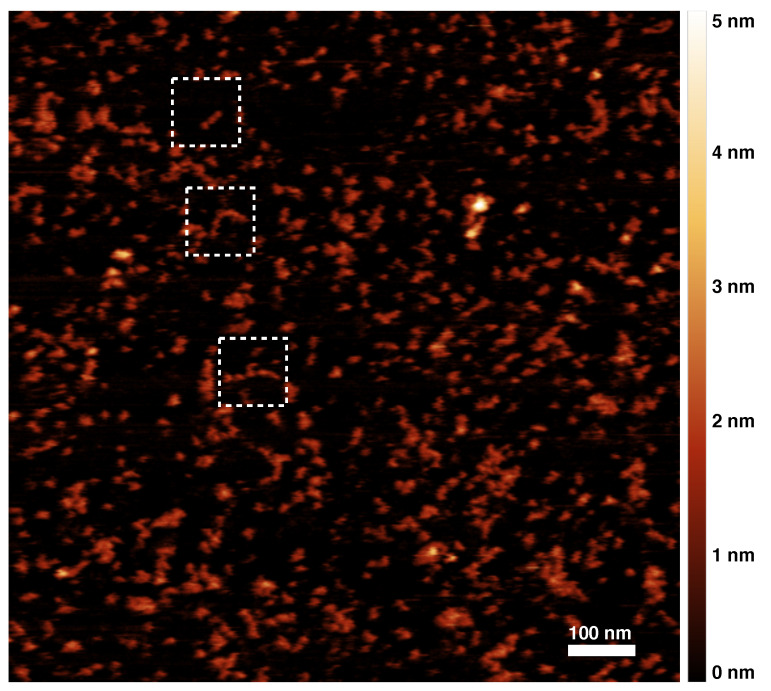
AFM image of a sample comprising Z $\alpha_{1}$AT monomers and polymers; the colored scale on the right indicates the height of the pictured objects and the dashed squares show 100 nm × 100 nm regions of interest analysed in Figure 3.

**Figure 2:**
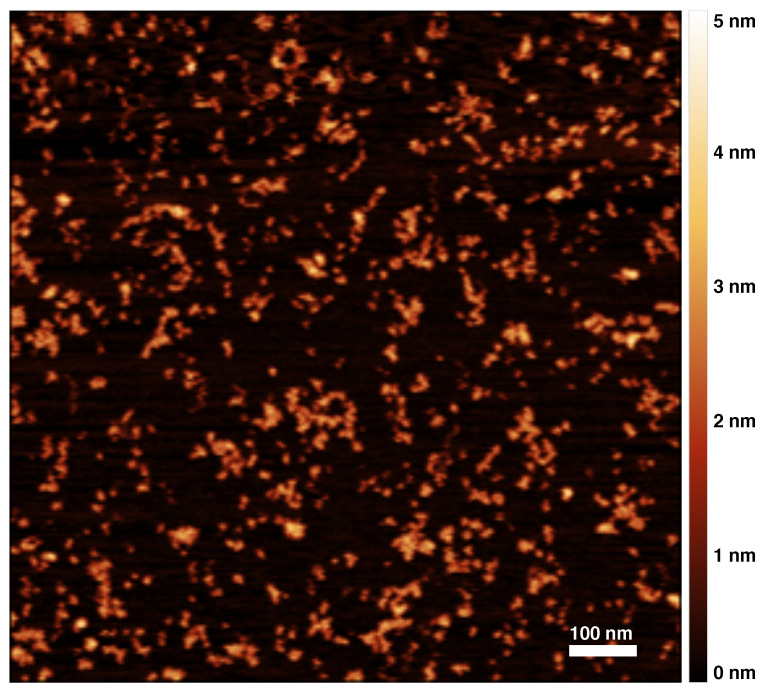
AFM image of M $\alpha_{1}$AT monomers and polymers; the colored scale on the right indicates the height of the pictured objects.

Figure 1 shows an AFM image of a sample of Z $\alpha_{1}$AT polymers. Other images are reported in the Supplementary Materials (Supplementary Figure S2). Images of bare monomeric samples did not show any tendency to coalesce due to deposition on the substrate. Monomers and polymers were identified by careful visual inspection, and their structural features were determined using the Gwyddion software package (Supplementary Figure S3). The actual quantities were corrected by taking into account the tip shape and size. While any deconvolution of tip effects can inevitably introduce some artefacts, in the collected images, the objects are sufficiently separate to allow us to apply the standard deconvolution algorithm of a parabolic tip on a step [33,34] and to use the expression: $w=w^{\prime }-2{\left(2hR_{tip}\right)}^{0.5}$, where $w^{\prime }$ and *w* are the apparent and deconvoluted widths, h is the step height of the object, and $R_{tip}$ is the tip radius of curvature. Analogous imaging and analysis were performed on M $\alpha_{1}$AT polymers, as displayed in Figure 2. The shapes and dimensions in the two samples were found to be qualitatively and quantitatively similar.

In parallel, we developed an Optimised Software for AFM Map Analysis (OSAMA), implemented in the GNU-octave environment [35]. The software purpose is to distinguish polymers from other objects in AFM images by means of an unsupervised and unbiased procedure and then to obtain the relevant properties of the polymers. At first, a custom script identifies the edges of each object in the AFM maps; the binary maps obtained are then processed to measure the main structural parameters, including the end-to-end distance and the contour length (Supplementary Figure S4). Both visual inspection and the software method gave consistent, if not identical, results (Supplementary Figure S5). A few structures in AFM images resembled cyclic or branched polymers. $\alpha_{1}$AT polymers are mediated by a domain swap that involves a specific structural change in a central β-sheet [9,14,18,19,36], and a self-propagating polymer chain achieved in this manner is only able to support unbranched growth. Therefore, we can assume that any observable crossing structure was due to the deposition process. Nevertheless, in a two-dimensional image, there is no actual means to distinguish whether a structure is branched, circularised, or superimposed; thus, all such ambiguous objects were discarded in the analysis.

The average values for the heights of monomers and polymers, the two main monomer axes of monomers, and the average width of polymers along their elongation are reported in Table 1. The actual measured dimensions have an intrinsic uncertainty due to the deconvolution procedure. Additionally, the measure of height is typically affected by the peculiar vertical interaction of the AFM tip with biological samples, which usually leads to underestimation of the true height. This notwithstanding, there is excellent agreement of our deconvoluted parameters with existing data. Indeed, crystallographic, small angle X-ray and neutron scattering data reported dimensions of 7.8 nm × 4.9 nm × 2.2 nm for native [37] and recombinant $\alpha_{1}$AT [38].

It is worth noting that the calculated height of the polymer is similar to that of the monomer, thus showing that the polymers are on average deposited as a single layer on the surface without crossover. The observation that the polymer width $w_{p}$ is lower than the major axis of the monomer $a_{m}$ (see Table 1) suggests that there is an angle between the major axis of the polymer subunits and the polymer path, in tune with recent high-resolution imaging of $\alpha_{1}$AT polymers [18].

**Figure 3:**
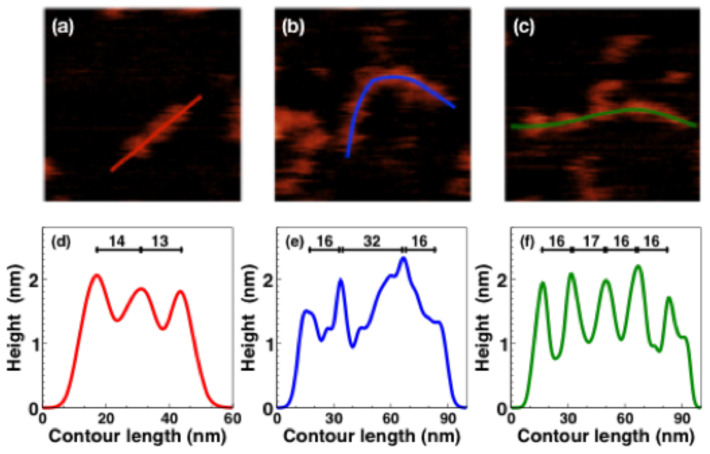
(**a**–**c**): Zoom of three regions of 100 nm × 100 nm size, selected from dashed squares in Figure 1. The elongation axis, or contour profile, of polymers is marked with colored solid curves. (**d**–**f**): height profiles along the contour length of polymers displayed in (**a**–**c**) respectively. The solid bars within each panel report the pitches between two peaks in the height profile; the numbers above each bar are the length in nanometres.

Figure 3 shows details from a few areas of the wide-field image (Figure 1). The height profiles reveal oligomers with pitches that typically vary between 14 nm and 16 nm, as seen in Figure 3d–f, These units, namely the distance between height maxima in the AFM maps, are inconsistent with the expected major axis length of a single subunit within the polymer. This distance is consistent with a maximally extended dimeric subunit but, given the evident polymer flexibility, most likely corresponds to multimer subunits with only a partial exposure to the AFM-accessible surface due to a slight twist. Consistently, some polymer profiles, such as in Figure 3e, show a finer structure, with secondary maxima at a shorter distance, thus confirming that the most evident pitch of 16 nm spans a unit of a few proteins. One may argue if analogous considerations may apply to previous imaging of $\alpha_{1}$AT polymers by AFM or EM [17,18,39,40].

**Figure 4:**
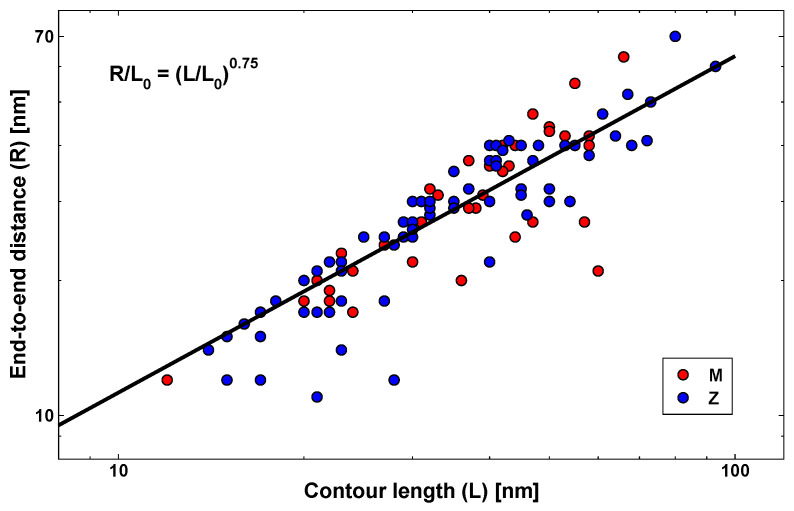
End-to-end distance (R) versus contour length (L) for M (red circles) and Z (blue circles) $\alpha_{1}$AT. The solid line represents a power law with exponent 0.75.

From AFM images, one can also measure the length of the polymers, or contour length *L*, along with the end-to-end distance *R*, which are displayed in Figure 4. Both *R* and *L* have been considered after deconvolution of the tip effect. They correlate with a power law having an exponent of 0.75. In particular, the best fit is given by the expression $R/L_{0}={\left(L/L_{0}\right)}^{\nu }$. The exponent ν describes how the size of a self-similar polymer chain increases. The power law is reminiscent of the scaling behaviour exhibited by long Gaussian polymer chains in classic polymer theory. In particular, in the case of a random polymer in a good solvent, Flory provided an elegant mean-field derivation of the exponent ν as a function of the space dimension *d*: ν=3/(d+2) [26,41]. In the case of a polymer in a planar surface, one has d=2 and ν=0.75, matching the current findings for $\alpha_{1}$AT polymers. The parameter $L_{0}$ is the Kuhn length, that is the length of a single unit in an ideal polymer chain, corresponding to double the persistence length [42]. In the present case, one obtains $L_{0}/2=8$ nm, eliciting a very high polymer flexibility. Gaussian polymer theory cannot be automatically applied to small protein polymers, such as those observed in the present work, without further evidence. On the other hand, it is interesting to consider this analogy.

Another scaling property was observed by using a different technique: light scattering. Highly concentrated solutions of purely monomeric M $\alpha_{1}$AT were incubated at high temperatures (either 55° or 70°), and the progress of polymerisation was monitored continuously by light scattering. We measured simultaneously the z-averaged hydrodynamic radius $R_{h}$ and the excess Rayleigh ratio $R_{ex}$ of polymer aggregates. The hydrodynamic radius $R_{h}$ is proportional to the average diffusion relaxation time τ and was determined from the autocorrelation functions of scattering intensity analysed by the cumulant method or, additionally, the central moment method (Supplementary Figure S6) [31]. The use of a second fitting procedure was considered to rule out any weakness of the cumulant method, since during the course of the polymerisation kinetics we obtained a relatively high polydispersity index, PDI, or equivalently a high normalised variance $\sigma^{2}$. We found that both analyses gave the same results and the distribution of polymer hydrodynamic radii were almost completely described by the first two cumulants. (Supplementary Figure S6). Interestingly, we observed that polymers grow uniformly with their average size always proportional to the width of the size distribution, or in other terms, with a constant normalised variance (or PDI) [22]. This implies that the distribution of polymer hydrodynamic radii exhibit a self-similar shape over the course of the kinetics (Supplementary Figure S7).

**Figure 5:**
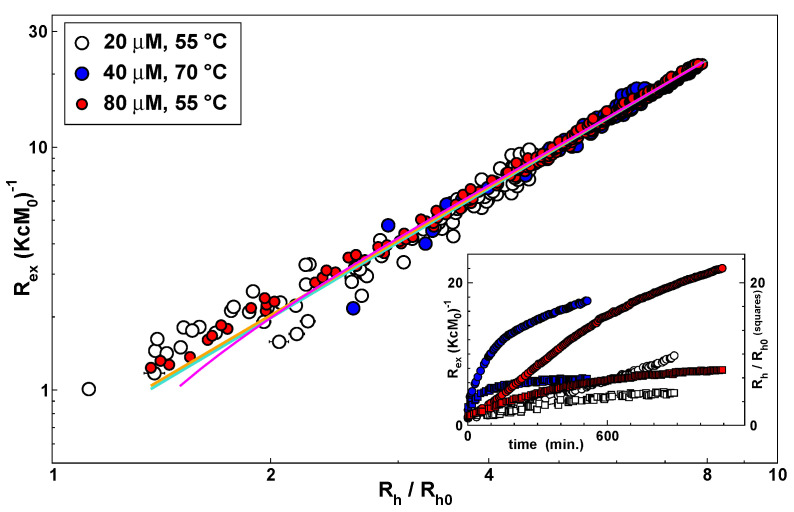
Normalised Rayleigh ratio, $R_{ex}{\left(KcM_{0}\right)}^{-1}$, vs. hydrodynamic radius, $R_{h}$, of M $\alpha_{1}$AT incubated at different temperatures and concentrations, as shown in the legend. Inset: polymerisation kinetics monitored over time in terms of $M_{w}$ (circles) and $R_{h}$ (squares). The orange solid line represents a power law with exponent 1.75. The green solid line represents a fit by using scaling expressions as described in the text. The magenta solid line represents a curve drawn by using WLC expressions as described in the text.

The self-similarity of the distributions of radii were mirrored by a scaling behaviour observed in the growth of the apparent mass. Indeed, Figure 5 shows the correlation between $R_{ex}{\left(KcM_{0}\right)}^{-1}$ and $R_{h}/R_{h0}$, where $M_{0}=52$ kDa and $R_{h0}=2.6$ nm are the molecular mass and the hydrodynamic radius of the monomers, respectively. As observable in the figure, at the onset of the kinetics, the apparent weight average mass $R_{ex}{\left(Kc\right)}^{-1}$ has a value close to the monomer mass $M_{0}$, encouraging us to rule out any interaction effect due to protein or polymer concentration. This is consistent with a recent NMR study in which concentrations of 20 mg/mL were found not to show evidence inter-monomer association [43]. Again, a powerlaw describes this correlation: $R_{ex}/\left(KcM_{0}\right)={\left(R_{h}/R_{h0}\right)}^{d_{h}}$, with $d_{h}=1.75$ (solid orange line in Figure 5). Remarkably, the scaling behaviour roughly extended to very small polymers, as also found with neuroserpin [22].

The scattered intensity was proportional to both the average mass $M_{w}$ and form factor P(q): $R_{ex}/\left(KcM_{0}\right)=M_{w}/M_{0}P\left(q\right)$, assuming that the monomer form factor is 1 at the observed scattering vector. At the end of the observed polymerisation time-course, we measured the form factor P(q) and the relaxation time τ(q) as a function of scattering vector *q* (Supplementary Figure S8). From these experiments, we obtained $M_{w}/M_{0}=34$, $R_{g}=36$ nm and $R_{h}=24$ nm. The ratio $R_{g}/R_{h}=1.5$ is typical of flexible polymer chains (Supplementary Figure S9). In the course of the kinetics, the form factor was not measured. Nevertheless, by following the analogy with flexible long chains, we may assume other scaling relations between the mass and the hydrodynamic radius ($M_{w}/M_{0}={\left[R_{h}/R_{h0}\right]}^{d_{h}}$) and the mass and the radius of gyration ($M_{w}/M_{0}={\left[R_{g}/R_{g0}\right]}^{d_{f}}$), and we may approximate the form factor with the expression $P{\left(q\right)}^{-1}=\left[1+{\left(qR_{g}\right)}^{2}/3\right]$, valid for $qR_{g}<1$ [44]. By using the latter expressions, one may interpolate the data in Figure 5, obtaining $d_{h}=1.75$ (solid green line in Figure 5).

Different arguments could be taken to rationalise a scaling relation between the mass and the hydrodynamic radius of polymers, including the physics of the aggregation process [44] or the shape factors. For instance, in the classic worm-like chain (WLC) model polymers are represented as flexible cylinders of diameter *d* and length *L*. The flexibility of the chain is described by the persistence length, $l_{p}$, or equivalently the Kuhn length, $l_{K}=2l_{P}$. A relation between the length of the chain, or its mass *M*, and the hydrodynamic radius $R_{h}$ can be calculated as a function of *d* and $l_{K}$ [45]. The Kratky–Porod expression for a semi-flexible chain can be used to calculate the radius of gyration as a function of *L* and $l_{K}$ [42]. In the limit of a long chain, the behaviour of a random polymer is recovered. It is interesting to note that, for small persistence lengths, a power law is soon recovered for both $R_{g}$ and $R_{h}$, while the exponents are not related to any universal behaviour (Supplementary Figure S9). As an example, a curve related to a worm-like chain with diameter equal to the hydrodynamic diameter $d=2R_{h}$ and $l_{K}/d=5$ is shown in Figure 5 (solid magenta curve), exhibiting a good correspondence with experimental data. In such a case, the total persistence length of the polymers is calculated to be $l_{p}=13$ nm, of the same order of monomer size and approaching the value obtained from the AFM analysis.

## 4. Conclusions

Aberrant polymerisation of mutant serpins is at the basis of a class of conformational diseases, the serpinopathies [46]. The design of molecules able to prevent polymerisation or to ameliorate its consequences represents a therapeutic strategy against the accumulation-related phenotypes associated with these diseases [47,48,49]. A diagnostic reporter probe that allows for the noninvasive evaluation of polymer load also represents a useful tool in the management of the pathologies associated with polymer accumulation. An important step towards the development of strategies is clarification of the structural details of polymers and the mechanisms of their formation [18,50].

Here, we applied AFM and LS to map the structural details of $\alpha_{1}$AT polymers, both for wild-type M polymers formed by heating and for Z variant polymers purified from patients. Our analysis provides a monomer size distribution in keeping with higher resolution studies using X-ray diffraction [37,38] and with a more recent experiments by AFM [17]. It has been shown using a conformationally selective antibody that ex vivo liver polymers present an epitope that is also found on heat-induced polymers [25]. The structural details shown here further extend the features that these two polymer forms hold in common. This further validates the use of heat induction in the study of polymer formation as shown in a number of studies [13,18].

**Figure 6:**
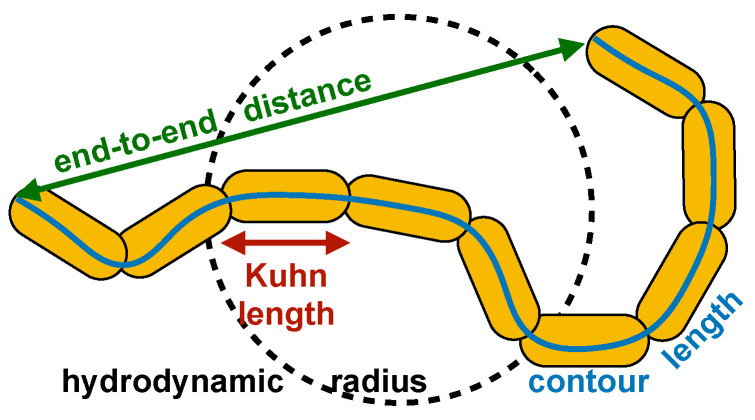
Scheme of polymer structural parameters. The cartoon sketches the main structural parameters identified in the experimental work, namely the end-to-end distance (green solid arrow), the contour length (blue solid curve), the hydrodynamic radius (black dashed circle), and the Kuhn length (red solid arrow).

In addition, our data exhibit a power law in the relationship among some structural parameters, namely the variation of end-to-end distance with contour length and the relationship between hydrodynamic radius and the polymer mass. The structural parameters relevant for the above analysis are sketched in the cartoon of Figure 6. This indicates that the polymer size scales with the number of polymer units and that such a scaling behaviour closely resembles that of a linear random chain in a good solvent as well as a flexible worm-like chain. Given the relatively small number of subunits present in serpin *protein* polymers when compared with polymers as defined in the chemical sense, according to some interpretations of classical polymer theory, these objects should exhibit a more rod-like behaviour. It is therefore interesting to consider the underlying structural basis for this apparent discrepancy. One answer to this may lie in the observation that these molecules are dual in nature: while comprised of large, rigid, and well-folded repeating serpin domains, their inter-domain linker consists of a stretch of more classically described *chemical* polymer subunits 5–10 amino acids in length. This latter element, in the absence of restraints imposed by subunit inter-surface polar and non-polar interactions, would confer a very high degree of orientational freedom. Indeed, the deviation from a rod-like behaviour allows us to draw the conclusion that there is a pronounced flexibility in this region barely if at all tempered by a consistent interface operating to restrict polymer conformation. This distinguishes $\alpha_{1}$AT polymers from the fibrillar amyloid structures, which provide unique intermolecular interfaces for binding of small reporter molecules. In regard to the development of diagnostic reagents capable of selectively binding to polymers in vivo, this suggests that a small molecule capable of recognising an intramolecular conformational adjustment associated with polymerisation would be more tractable than one relying on presentation of a well-formed intermolecular interface.

Our analysis shows a persistence length comparable to the length of a monomer, highlighting the high flexibility of $\alpha_{1}$AT polymers. Such a flexibility gives a rationale for the observation of scaling behaviour in such small objects, even down to the monomer size.

The use of scaling concepts in polymer physics, as in the classical text book by deGennes [26], has a long-standing tradition for different kind of polymers [42], including protein aggregates and amyloid fibrils [22,44]. Here, we frame the studies of serpin polymers within classical polymer physics and foster a complementary perspective to the mainstream research in the structural biology of serpin polymers.

## Figures and Tables

**Table 1:** Average sizes from the analysis of Figure 1 and Figure 2. $h_{m}$, monomer height; $a_{m}$, monomer major axis; $b_{m}$, monomer minor axis; $h_{p}$, polymer height; $w_{p}$, polymer width (orthogonal to the elongation axis).

	$h_{m}$	$a_{m}$	$b_{m}$	$h_{p}$	$w_{p}$
**Z $\alpha_{1}$AT**	1.8 ± 0.3 nm	7.8 ± 1.6 nm	3.2 ± 0.7 nm	1.8 ± 0.2 nm	5.3 ± 0.8 nm
**M $\alpha_{1}$AT**	1.8 ± 0.5 nm	9.3 ± 1.5 nm	3.1 ± 0.6 nm	1.9 ± 0.4 nm	5.4 ± 1.0 nm

## Data Availability

Data are available on request from the corresponding author.

## Supplementary Materials

The following are available online at https://www.mdpi.com/article/10.3390/ma14102577/s1. Figure S1: Non denaturing PAGE; Figure S2: Supplementary AFM images; Figure S3: AFM statistical analysis; Figure S4: Optimised Software for AFM Map Analysis (OSAMA); Figure S5: Validation of the unsupervised procedure used by the OSAMA software; Figure S6: Dynamic light scattering analysis; Figure S7: Polydispersity index (PDI) during polymerisation kinetics; Figure S8: Form factor and diffusion of $\alpha_{1}$AT polymers; Figure S9: Application of the worm-like chains (WLC) model.
