# Supplementary material for: Scaling Concepts in Serpin Polymer Physics
Source: Materials (Basel). 2021 May 15;14(10):2577. doi: 10.3390/ma14102577 (PMC8156723; doi:10.3390/ma14102577)
Supplement: Supplementary file 1 [file materials-14-02577-s001.zip › materials-14-02577-s1.pdf]

Supplementary Materials: Scaling Concepts in Serpin Polymer Physics

Samuele Raccosta [1], Fabio Librizzi [1], Alistair M. Jagger [2,3], Rosina Noto [1], Vincenzo Martorana [1], David A. Lomas [2,3], James A. Irving [2,3], Mauro Manno [1,*]

Index of supplementary figures

- Figure S1: Non denaturing PAGE
- Figure S2: Supplementary AFM images
- Figure S3: AFM statistical analysis
- Figure S4: Optimised Software for AFM Map Analysis (OSAMA)
- Figure S5: Validation of the unsupervised procedure used by the OSAMA software
- Figure S6: Dynamic light scattering analysis
- Figure S7: Polydispersity index (PDI) during polymerisation kinetics
- Figure S8: Form factor and diffusion of α_1AT polymers
- Figure S9: Application of the Worm-like chains (WLC) model

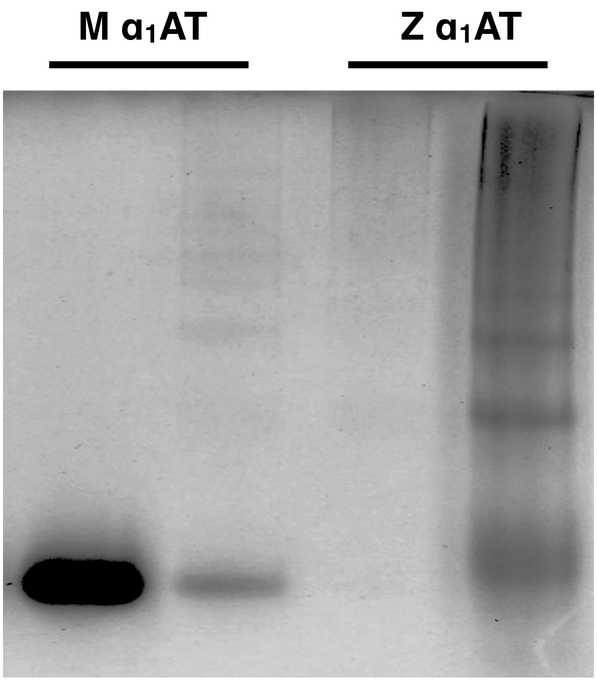

Figure S1. Non denaturing PAGE (polyacrylamide gel electrophoresis) of α_1AT monomers and polymers. Lane 1: M-α_1AT monomer; lane 2: M-α_1AT polymer; lane 3 & 4: Z-α_1AT polymer with different levels of loading.

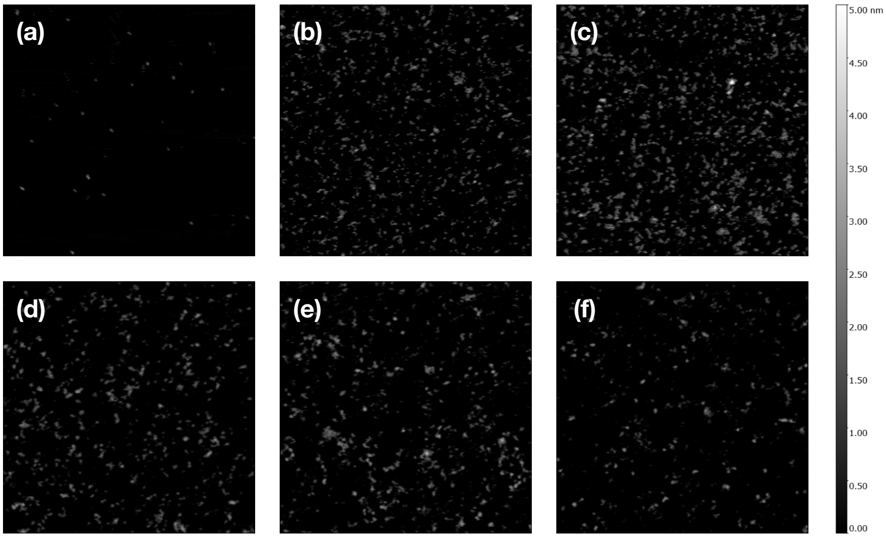

Figure S2. Supplementary AFM images of α_1AT monomers and polymers. (a) M α_1AT monomers; (b)-(c) Z α_1AT polymers; (d)-(f) M α_1AT polymers;

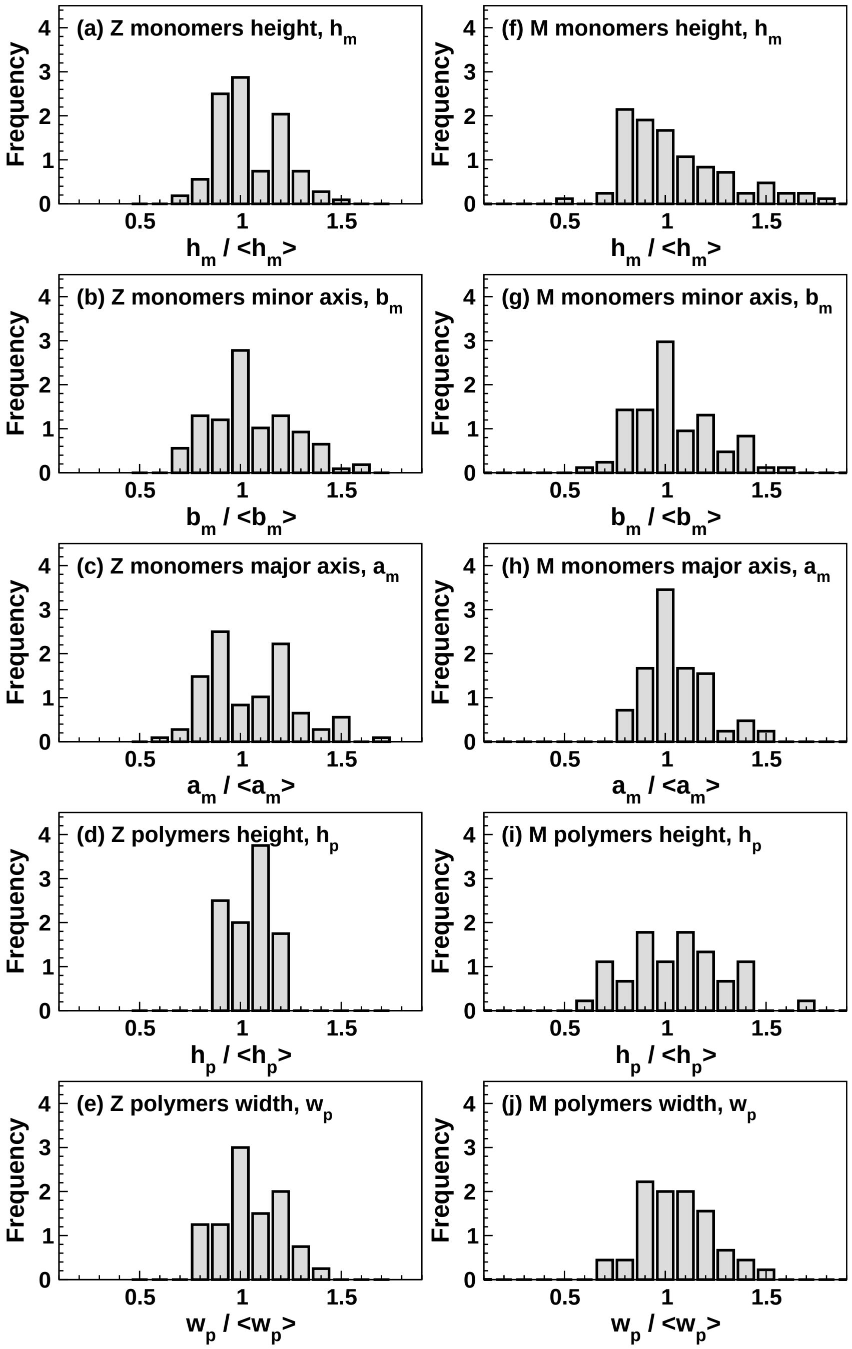

Figure S3. AFM statistical analysis for some features of monomers and polymers of Z α_1AT, left panels (a)-(e), and M α_1AT, right panels (f)-(j). (a),(f) monomers height h_m; (b),(g) monomers minor axis b_m; (c),(h) monomers major axis a_m; (d),(i) polymers height h_p; (e),(j) polymers width orthogonal to the contour length w_p.

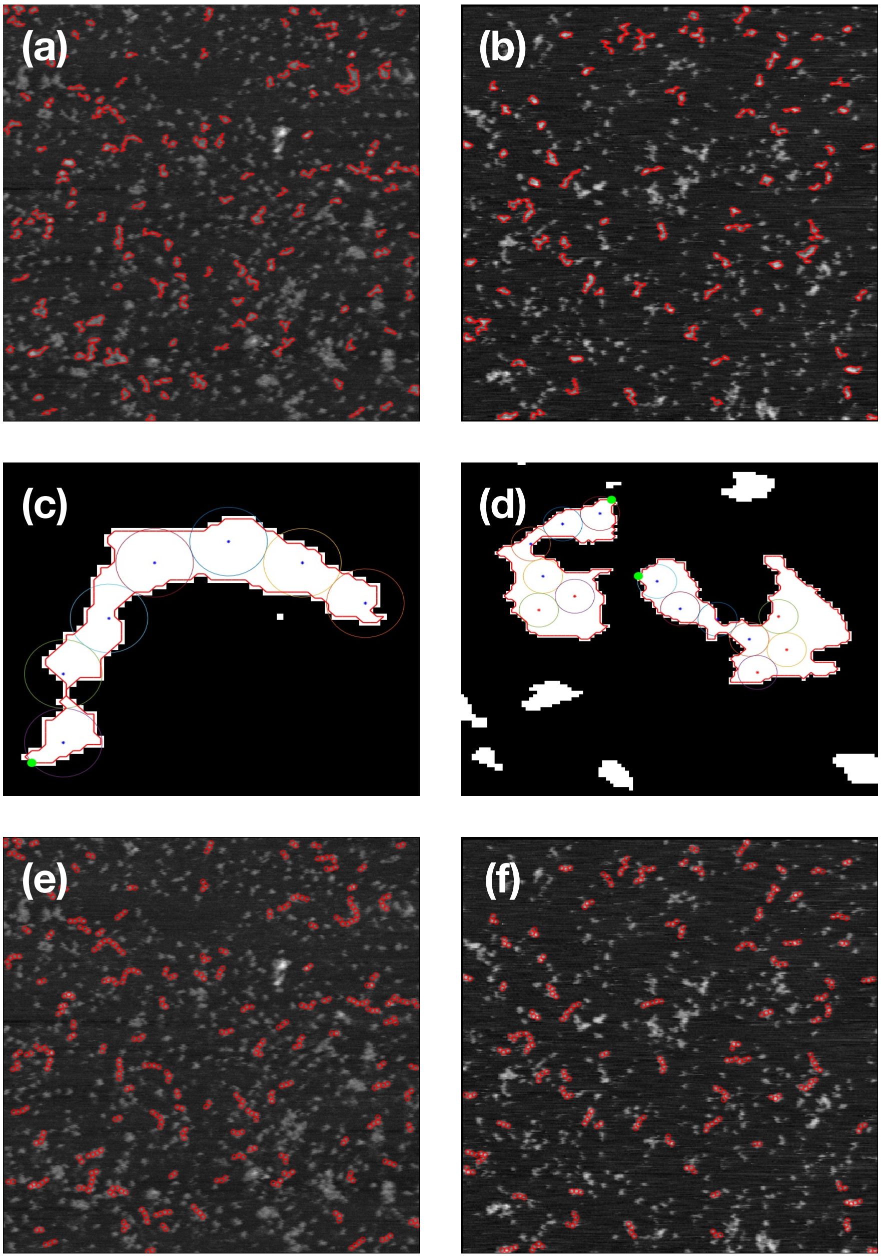

Figure S4. Optimised Software for AFM Map Analysis (OSAMA).
The panels highlight the different steps of software procedure. As a first step, the images were rendered in black and white by using a threshold cut-off as in panel (a) for Z α_1AT and panel (b) for M α_1AT, allowing to identify the edges of each object (red contour lines). Then, the software attempts to cover each object by using a series of consecutive circles, as shown in panels (c) and (d). The covering procedure starts from one extreme of the object, chosen as the point for which the momentum of inertia of the object results maximum: the green dots in panels (c) and (d). Afterwards, circles are added one by one, by maximizing in each step the overlap between the object and the circle. As long as this procedure success in covering the object without any ambiguity, as in panel (c), the object is recognized as a polymer ("successful polymer recognition"). At the opposite, the object is rejected when the procedure is unsuccessful, as shown in panel (d), where the covering may proceed in more the one direction ("unsuccessful polymer recognition"). The radius of the circles used (5.5 nm) results from a compromise between the requirements of a good overlap when covering the objects and the efficiency in the rejection of objects which undoubtedly are not polymers. It is also compatible with the thickness of the objects clearly appearing as polymers, as shown in panel (e) for Z α_1AT and panel (f) for M α_1AT, where each recognised polymer is covered with the sequence of red circles.

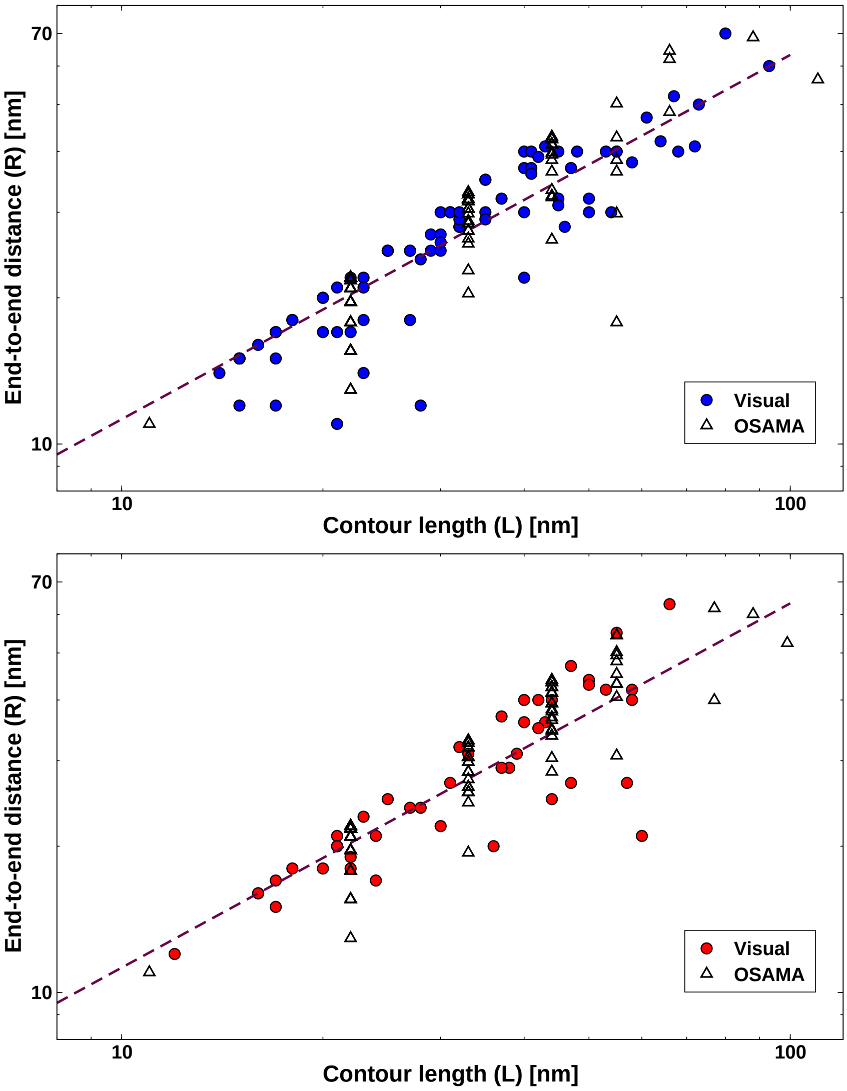

Figure S5. Validation of the unsupervised procedure used by the OSAMA software.
A comparison is shown between the analysis performed by the OSAMA script and by visual inspection for Z α_1AT polymers (upper panel) and M α_1AT polymers (lower panel). For the objects recognized as polymers, the end-to-end distance and the contour length can be estimated in a first approximation by using the position of the centers of the first and the last circles. This choice may probably lead to a slight underestimation of both parameters, but has the advantage of being unequivocally determined. Furthermore, as AFM images were convoluted for the tip curvature, the underestimation of both parameters may be much smaller than the radius of the circles. The contour length values obtained by OSAMA are discrete, being a multiple of the diameter of the circles used for the covering. Nevertheless, the two sets of data result in very good agreement.

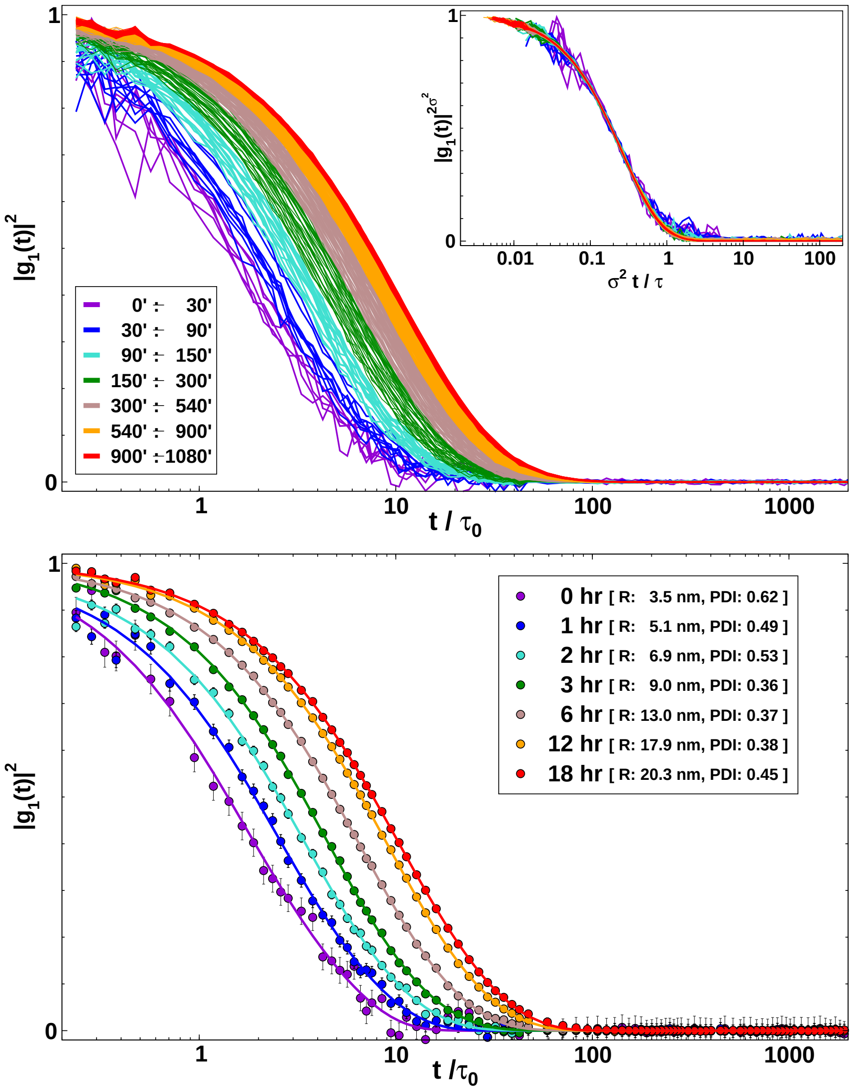

Figure S6. Dynamic light scattering analysis.
Upper panel. Autocorrelation functions $|g_1(t)|$ during polymerisation of a 80 μM solution at 55 °C. The delay time t, originally in the μs range, is normalised by the diffusional relaxation time τ_0 of a particle with a hydrodynamic radius of 1 nm. Different kinetic times are identified by different colours, as in the legend. *Inset*: functions of the main panel rescaled by using the average relaxation time τ and the normalised variance σ^2, related to the first ($k_1 = \tau^{-1}$) and the second cumulant ($k_2 = \sigma^2\tau^{-2}$) in the expansion of autocorrelation functions: $\ln[g_1(t)] = -t/\tau + 1/2\sigma^2[t/\tau]^2 + O([t/\tau]^3)$.
Lower panel. Selected autocorrelation functions at different times over the course of the kinetics (as specified in the legend). The curves were fitted by using the central moments analysis: $|g_1(t)| = e^{-t/\tau}[1 + 1/2\sigma^2[t/\tau]^2 + O([t/\tau]^3)]$. The legend also reports the average hydrodynamic radius R and the polydispersity index (PDI=σ^2).

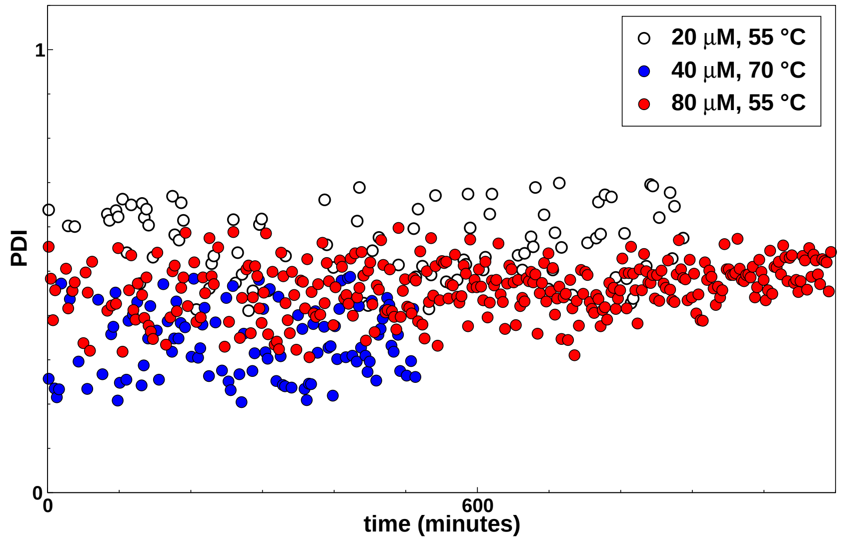

Figure S7. Polydispersity index (PDI) during polymerisation kinetics.
PDI is measured as the normalised variance σ^2, that is the ratio between the second cumulant k_2 and the square of the first cumulant k_1^2. The average PDI values are 0.46 ± 0.06, 0.34 ± 0.08 and 0.57 ± 0.08, for 80 μM, 40 μM and 20 μM, respectively. Within experimental error, the normalised variance remained constant over the course of the kinetics.

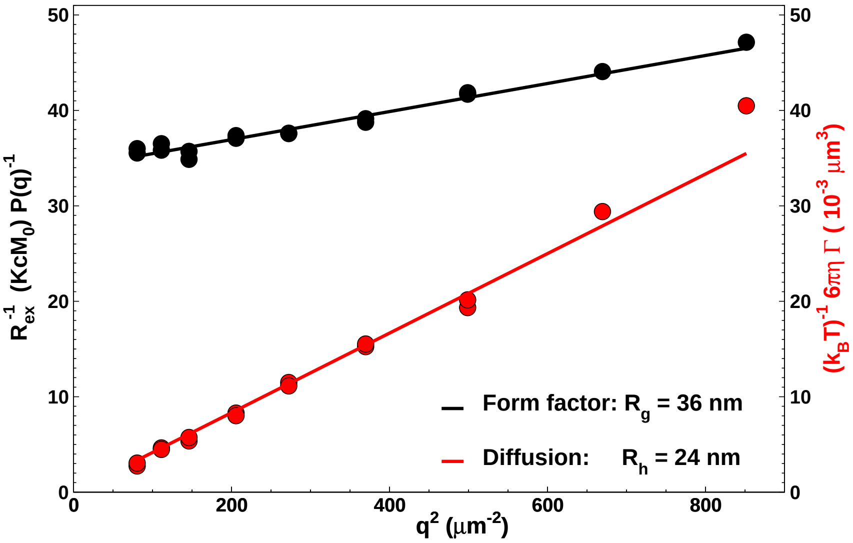

Figure S8. Form factor and diffusion of α_1AT polymers.
Normalised form factor (black circles) and normalised diffusion relaxation time (red circles) of M α_1AT polymers formed by heating a 80 μM solution at 55 °C overnight. The black solid lines show a fit to the data: $(KcM_0)^{-1}R_{ex}P(q) = M_w/M_0\left[1+\frac{1}{3}q^2R_g^2\right]^{-1}$, with $M_w/M_0 = 34$, and $R_g = 36nm$. The red solid line is a fit to the data: $k_BT(6\pi\eta\Gamma)^{-1} = R_hq^{-2}$, with $R_h = 24nm$.

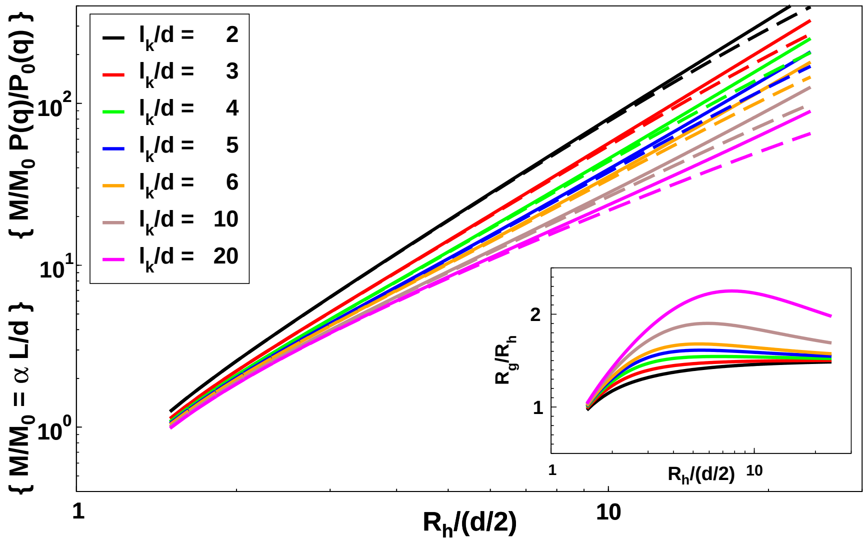

Figure S9. Application of the worm-like chain model (WLC).
In this model, the polymer is represented as flexible cylinder of diameter d and length L. The flexibility of the chain is described by the persistence length, l_p, or equivalently the Kuhn length, $l_K = 2l_P$. The polymer mass M is proportional to the polymer length $M/M_0 = \alpha L/d$, where M_0 is the protein mass and α is a coefficient which is close to the ratio between d and the major axis of the protein monomer ($\alpha =$ 0.6). Coloured solid lines display the relation of M/M_0 against the polymer hydrodynamic radius R_h for different values of l_K/d, calculated by using the expression of Yamakava and Fujii (Macromolecules 1973, 6, 407-415). Coloured dashed lines display the correction to M/M_0 due to the polymer form factor $P(q)/P_0(q)$, where $P_0(q)$ is the monomer form factor and $q = 22.33\mu m^{-1}$ is the scattering vector used in the present work. The form factor was calculated using the expression: $P(q)^{-1} = 1 + \frac{1}{3}q^2R_g^2$, where R_g is the radius of gyration. The R_g was calculated by using the Kratky-Porod expression for semi-flexible worm-like chains (see, e.g., Doi and Edwards, The theory of Polymer dynamics, 1986): $R_g^2 = \frac{l_K^2}{12}\left[\frac{L}{l_K} - 1 + \mathrm{e}^{-\frac{L}{l_K}}\right]$. The inset displays the ratio between R_g and calculated by using the above expressions at different values of l_K/d.
